# Role of Plasma Calreticulin in the Prediction of Severity in Septic Patients

**DOI:** 10.1155/2019/8792640

**Published:** 2019-09-12

**Authors:** Zhiheng Xu, Yuanyuan Yang, Jianmeng Zhou, Yongbo Huang, Ya Wang, Yu Zhang, Yuan Lan, Jie Liang, Xiaoqing Liu, Nanshan Zhong, Yimin Li, Pu Mao

**Affiliations:** State Key Laboratory of Respiratory Disease, National Clinical Research Center for Respiratory Disease, Guangzhou Institute of Respiratory Health, First Affiliated Hospital of Guangzhou Medical University, Guangzhou Medical University, Guangzhou 510120, China

## Abstract

**Background:**

Calreticulin has been identified to play a critical role in innate and adaptive immune responses. However, little is known about the role of calreticulin in sepsis with a characteristic of immune disorder. This study was aimed at investigating whether plasma calreticulin level increases in sepsis and its association with sepsis severity.

**Methods:**

This retrospective analysis evaluated sepsis patients who were admitted to the intensive care unit (ICU). Healthy subjects were also included as controls. Plasma samples were collected from the patients within 48 h after ICU admission as well as the healthy subjects. Plasma calreticulin levels were measured via the enzyme-linked immunosorbent assay.

**Results:**

In total, 127 sepsis patients and 40 healthy controls were included. Calreticulin was significantly increased in sepsis patients than in healthy controls. Furthermore, the level of plasma calreticulin was significantly higher in nonsurvivors than in survivors. Patients with calreticulin levels > 343.5 pg/ml showed lower cumulative survival than those with levels < 343.5 pg/ml.

**Conclusion:**

Calreticulin level was positively correlated with the severity of sepsis. High calreticulin level indicated poor prognosis of sepsis patients.

## 1. Introduction

Sepsis is a clinical syndrome associated with a systemic response to infection and an overexuberant inflammatory response, leading to profound cell and organ injury [1]. Despite improvements in intensive care unit (ICU) care during the last 15 years, sepsis remains a life-threatening condition, with mortality rates of 30%–50% [1, 2]. Both innate and adaptive immune system dysfunction play a crucial role during sepsis [3]. Sepsis could alter the innate and adaptive immune responses through immune suppression and chronic inflammation [4]. Immune cell apoptosis has been implicated to be an important mechanism of sepsis, and this is usually followed by multiorgan failure [5]. Thus, identifying a septic immune-related checkpoint of cell death may provide further insight into the pathophysiology of sepsis; further, these checkpoints may serve as predictors of sepsis severity.

Calreticulin is a calcium-binding chaperone that is normally an endoplasmic reticulum-resident protein [6], but it has also been identified to play a role in immunogenic cell death and extracellular functions [7]. Under certain stress conditions, including environmental, drug-induced, and hypoxia, calreticulin may be upregulated to regulate cell survival, death, or repair [7]. A previous study showed that calreticulin levels increase during lipopolysaccharide-induced apoptosis [8]. Calreticulin could be secreted from cells [9] and could bind directly to FasL to inhibit FasL-mediated apoptosis of Jurkat T cells [10]. Recent studies showed that calreticulin could be expressed on apoptotic cells and contribute to the phagocytosis of the dying cells [11–13]. In addition, calreticulin at higher concentrations in the plasma and synovial fluid of rheumatoid arthritis patients correlated with disease severity [10, 14]. Thus, calreticulin released into the extracellular space may play an important role in immunogenic cell death and inflammatory diseases, which are the key features of sepsis.

However, little is known about the role of calreticulin in sepsis in a clinical context. Thus, this study was aimed at investigating the plasma levels of calreticulin in sepsis patients and the potential correlations between plasma calreticulin levels and sepsis severity.

## 2. Methods

### 2.1. Patients and Ethical Concerns

This study was conducted in the Department of Intensive Care Unit of the First Affiliated Hospital of Guangzhou Medical University, from October 2012 to May 2015. We enrolled consecutive patients who fulfilled the sepsis criteria according to Sepsis-3.0 [1, 15]. The ICU patients were excluded if they underwent organ transplantation, have cancer, autoimmune disease, or human immunodeficiency virus (HIV), or are younger than 18 years. Healthy volunteers were also enrolled from our medical examination center as the control group. The study was approved by the Ethics Review Committee of the First Affiliated Hospital of Guangzhou Medical University (#201311), and written informed consent was obtained from patients or surrogates.

### 2.2. Study Design

Data on demographic characteristics (age and sex), laboratory test findings (blood count and arterial blood gas), length of ICU stay, and 28-day outcomes were collected from medical records. Disease severity was assessed using the Acute Physiology and Chronic Health Evaluation II (APACHE II) score. The primary endpoint was the 28-day sepsis-related mortality. The secondary aim was the relationship between calreticulin levels and sepsis severity.

### 2.3. Assay Procedures

Plasma calreticulin levels were measured using the enzyme-linked immunosorbent assay according to the manufacturer's instruction (Cusabio, CSB-E09787h). Plasma was collected within 48 h after ICU admission. Briefly, whole blood (5 mL) was drawn and centrifuged at 1000 rpm for 10 min, and the supernatant was stored at -80°C until use.

### 2.4. Statistical Analysis

Nonnormally distributed variables were expressed as the median and interquartile range (IQR). The Mann–Whitney *U* test was performed to compare continuous variables between survivors and nonsurvivors. Correlation was determined via the Spearman rank test. Receiver operating characteristic (ROC) curves were used to select the optimal cutoff value for calreticulin. Kaplan-Meier survival analysis using calreticulin groups as strata was conducted via log-rank tests. A univariate and multivariate Cox proportional hazards regression model was used to identify risk factors for 28-day mortality. All analyses were performed using SPSS 23.0 (SPSS Inc., Chicago, Ill.), and all figures were constructed using Prism 8.0 (GraphPad Software, La Jolla, Calif.). A two-sided *p* value < 0.05 was considered statistically significant.

## 3. Result

### 3.1. Study Population

**Figure 1:**
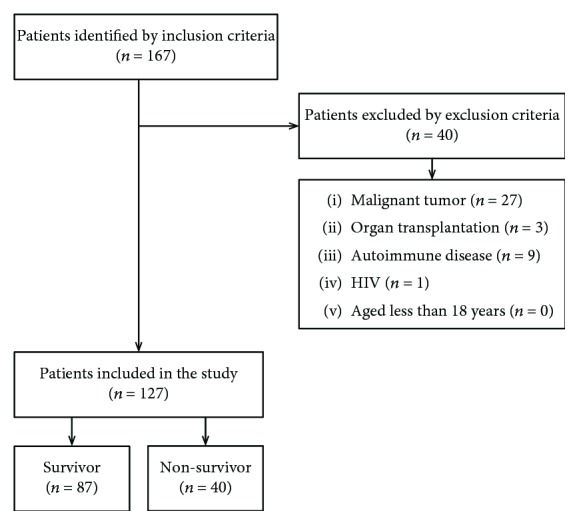
Selection of patients for inclusion in this analysis.

In total, 167 sepsis patients were screened for the study. Of them, 40 were excluded because they underwent organ transplantation (*n* = 3), had malignant tumors (*n* = 27), had autoimmune disease (*n* = 9), and had HIV (*n* = 1). Finally, 127 patients (93 males and 34 females) were followed up for 28 days. During the 28-day follow-up period, 40 patients died (Figure 1).

**Figure 2:**
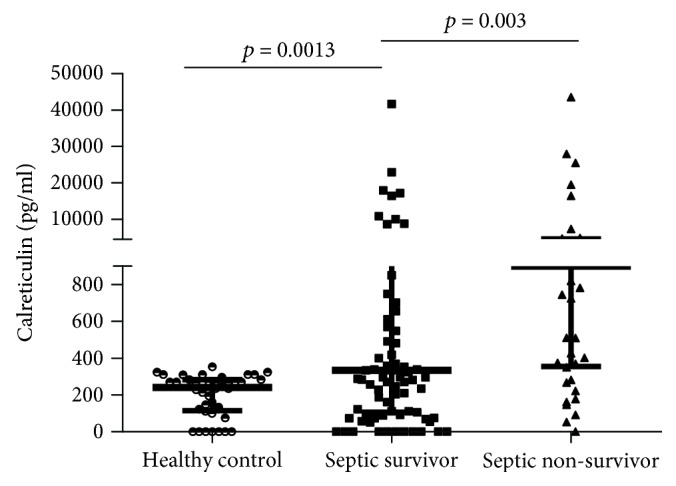
Plasma calreticulin levels.

The baseline clinical demographic data of the cohort are presented in Table 1. The control group comprised 40 patients; of these, 18 were men and 22 were women, and they matched for age with the study population (mean (SD) 63.0 ± 4.5 vs. 64.5 ± 14.5, *p* = 0.53). There was no significant difference in age or sex between sepsis survivors and nonsurvivors. However, the heart rate, respiration rate, mean arterial pressure (MAP), and PaO_2_/FiO_2_ were significantly different between the two groups (*p* < 0.05). The APACHE II score was significantly higher among nonsurvivors than among survivors (median (IQR): 25 (17-29) vs. 17 (13-22); *p* < 0.0001), while ICU stay was significantly shorter in nonsurvivors than in survivors (median (IQR): 8 (5-13) days vs. 25 (15-28) days; *p* < 0.0001). The level of plasma calreticulin was significantly higher in sepsis patients than in healthy controls (median (IQR): 418 (177-196) pg/ml vs. 240 (113-283) pg/ml; *p* < 0.0001; Figure 1). Further, plasma calreticulin was significantly increased in nonsurvivors compared to survivors (median (IQR): 899 (354-450) pg/ml vs. 333 (107-129) pg/ml; *p* = 0.03; Figure 2).

### 3.2. Correlations between Levels of Plasma Calreticulin and Other Clinical Parameters

Calreticulin levels were positively correlated with the APACHE II score, heart rate, respiration rate, and lactate levels (*p* < 0.05) (Table 2), but negatively associated with PaO_2_/FiO_2_ (*p* = 0.027) (Table 2).

### 3.3. Correlation between Calreticulin Levels and Sepsis Outcome

**Figure 3:**
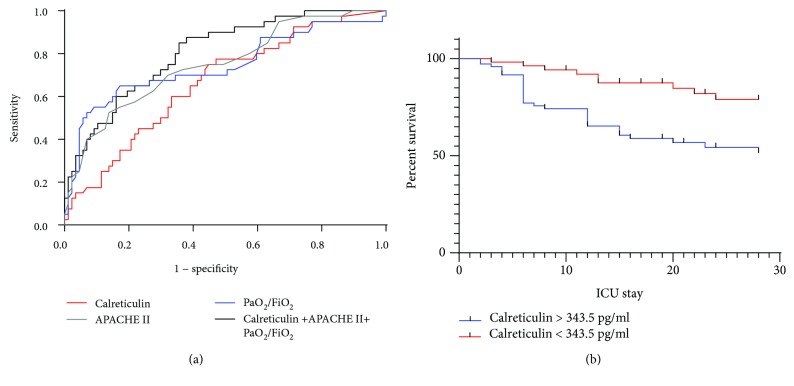
Analysis of sepsis mortality stratified by the calreticulin level.

Because of the correlation observed between calreticulin levels and sepsis severity, ROC curves and survival analysis were performed to investigate the predictive value of calreticulin for sepsis mortality. We found that plasma calreticulin levels could predict 28-day sepsis mortality, with an area under the ROC curve (AUC) of 0.664 (*p* = 0.003). In addition, using PaO_2_/FiO_2_ ratios and APACHE II scores to predict sepsis mortality, we obtained AUC values of 0.743 (95% CI, 0.64–0.85; *p* < 0.0001) and 0.749 (95% CI, 0.66–0.84; *p* < 0.0001), respectively. Combining the plasma calreticulin level with other markers would improve its AUC value [16]. The combined model using the calreticulin level, PaO_2_/FiO_2_, and APACHE II score showed better predictive capability, with an AUC of 0.805 (95% CI, 0.73–0.88; *p* < 0.0001; Figure 3(a)).

The optimal calreticulin cutoff value according to the best Youden index was 343.5 pg/ml, which had a sensitivity of 77.5% and a specificity of 52.9%. Sepsis patients were stratified according to calreticulin levels above and below 343.5 pg/ml. Notably, the Kaplan–Meier survival curves demonstrated that sepsis patients with calreticulin levels above 343.5 pg/ml were at a greater risk of death than others (Figure 3(b)).

In the univariate Cox regression model, parameters associated with 28-day mortality included respiration rate, MAP, PaO_2_/FiO_2_, WBC, PMN, lactate level, APACHE II score, and plasma calreticulin (*p* < 0.05) (Table 3). In the multivariable Cox proportional hazards model, after adjusting for age and sex, only APACHE II score (HR: 1.070; 95% CI: 1.014-1.129; *p* = 0.013) and respiration rate (HR: 1.141; 95% CI: 1.057-1.233; *p* = 0.001) at the time of admission were found to be independent predictors of 28-day ICU mortality (Table 3).

## 4. Discussion

Our study examined the correlation between baseline plasma calreticulin levels and the severity of sepsis. We found significantly higher plasma calreticulin levels in nonsurvivors than in both healthy persons and sepsis survivors. Moreover, we identified that increasing calreticulin levels was associated with higher mortality in sepsis patients.

The APACHE II score system developed in 1985, which is widely used to measure disease severity, has shown a positive correlation with hospital mortality and length of hospital stay [17]. However, its application for predicting disease progression and prognosis in sepsis is limited because it involves complicated calculations and subjective measurements that introduce ambiguities [18, 19]. Therefore, it is necessary to identify molecular predictors for disease severity, particularly in blood samples, that are easily accessible. In our study, we confirmed the significant positive correlations between the calreticulin value and APACHE II score. Moreover, plasma calreticulin levels were associated with increased risk of mortality in sepsis. These findings indicate that plasma calreticulin levels may very well complement the APACHE II score for assessing severity in sepsis patients.

For risk assessment, we identified the APACHE II score and respiration rate as independent risk factors for mortality for patients with sepsis. Plasma calreticulin level was correlated with sepsis mortality in the univariate Cox regression model. However, after adjusting for age and sex in the multivariate Cox regression model, we found that calreticulin level was not a significant risk factor for mortality. Sepsis is a heterogeneous and complex syndrome with various etiologies [20, 21]. This finding might suggest that to accurately predict the progression of sepsis, combining calreticulin level with other molecular markers is needed for the recognition of sepsis severity. In our ICU, the majority of patients were man, almost more than twice as many as woman. Considering this factor, prospective and multicenter observational studies will be needed to understand the exact value of calreticulin in predicting sepsis mortality.

PCT was one of conventional sepsis markers [22, 23]. In our study, we also detected PCT of sepsis patients. PCT was significantly higher in sepsis nonsurvivors than in survivors. However, PCT was not related to the severity of sepsis and death according to our data. In addition, calreticulin was correlated with the severity of sepsis according to our results. Calreticulin may be used as a predictor of sepsis mortality according to the following advantages. First, calreticulin was recognized as a multifunctional protein detected in apoptosis and innate and adaptive immune response [7, 24]. High levels of calreticulin are correlated with the severity of sepsis as proven in our study, which may reflect the active role of calreticulin in apoptosis and immune dysfunction in sepsis. Second, calreticulin levels in sepsis could provide important clinical information including sepsis severity that can be beneficial for clinical decision-making. Third, calreticulin may be a therapeutic target in sepsis. Neutralizing high levels of calreticulin in sepsis may provide a targeted treatment modality in sepsis.

The robustness of our study is supported by the following strengths. First, the sample size (*n* = 127) of the primary cohort was relatively large. Second, we excluded patients with autoimmune diseases who have been reported to have high levels of calreticulin [10, 25]. Third, collection of venous blood within 48 hours of ICU admission guaranteed that the levels of calreticulin were not yet to be affected by other treatment modalities.

However, there are also certain limitations in our study. First, we reviewed only the septic patients treated in our single center during a specific time period. For this reason, the sample size in our study was limited. A prospective and multicenter study with a larger sample size would be more convincing. Second, calreticulin levels of sepsis patients were measured only in the first 48 hours after admission. Continuous monitoring of plasma calreticulin levels would more precisely and thoroughly show a correlation between calreticulin and sepsis severity. Third, the pathophysiological role of calreticulin in sepsis remains to be completely understood. Animal models of sepsis are needed to further explore the mechanism by which calreticulin levels increase in sepsis.

## 5. Conclusions

Calreticulin level was positively correlated with the severity of sepsis, and increased calreticulin level indicated poor prognosis of patients with sepsis. Further studies on the role of calreticulin in sepsis are required.

## Figures and Tables

**Table 1:** Baseline patient characteristics.

Characteristics	Control group (*n* = 40)	All patients (*n* = 127)	Survivors (*n* = 87)	Nonsurvivors (*n* = 40)	*p* value
Age (years)	63 (60-66)	66 (54-76)	64 (52-75)	70 (60-80)	0.050
Sex (male/female)	18/22	93/34	63/24	30/10	0.761
APACHE II score	—	19 (15-25)	17 (13-22)	25 (17-29)	0.001
ICU stay (day)	—	19 (8-28)	25 (15-28)	8 (5-13)	0.001
Temperature	—	38.2 (37.7-38.7)	38.2 (37.8-38.2)	38.7 (37.1-38.7)	0.634
Heart rate	—	103 (94-117)	102 (90-115)	110 (99-120)	0.045
Respiration rate	—	23 (20-27)	22 (20-26)	25 (17.5-28.75)	0.002
MAP (mmHg)	—	80 (72.3-91.7)	82 (76-95)	75 (67-85)	0.007
WBC, 10^9^/L	—	12.8 (9.7-17.8)	12 (9.21-15.3)	16.6 (11.7-23.2)	0.001
PMN, 10^9^/L	—	11.2 (8.5-15.5)	10.2 (7.9-13.4)	14.7 (10.7-22)	0.001
PaO_2_/FiO_2_	—	177 (123-225)	194 (153-242)	110.5 (83-201.5)	0.001
Lactate (mmol/l)	—	1.7 (1.4-2.3)	1.61 (1.4-2.1)	2.1 (1.6-3.8)	0.003
PCT (ng/ml)	—	1.43 (0.33-10.91)	0.97 (0.29-8.55)	3.8 (0.85-12.6)	0.045
Calreticulin (pg/ml)	240 (113-283)	418 (177-196)	333 (107-129)	899 (354-450)	0.003

Data are expressed as medians (interquartile range). APACHE II score: Acute Physiology and Chronic Health Evaluation score II; ICU: intensive care unit; MAP: mean arterial pressure; WBC: white blood cell; PMN: neutrophils; PCT: procalcitonin.

**Table 2:** Correlations between baseline calreticulin and clinical parameters.

Parameters	*r* (Spearman rho)	*p* value
APACHE II score	0.200	0.024
Heart rate	0.182	0.04
Respiration rate	0.259	0.003
Lactate	0.291	0.001
PaO_2_/FiO_2_	-0.196	0.027

Calreticulin levels were positively correlated with the APACHE II score, heart rate, respiration rate, and lactate levels (*p* < 0.05), but negatively associated with PaO_2_/FiO_2_. APACHE II score: Acute Physiology and Chronic Health Evaluation II score.

**Table 3:** Cox proportional hazards models for mortality prediction based on calreticulin levels and severity scores.

Variable	Univariate Cox model	*p* value	Multivariate Cox model	*p* value
	HR (95% CI)		HR (95% CI)	
Heart rate	1.016 (1.000-1.033)	0.051	0.979 (0.957-1.000)	0.052
Respiration rate	1.098 (1.038-1.160)	0.001	1.141 (1.057-1.233)	0.001
MAP	0.972 (0.951-0.994)	0.014	1.001 (0.974-1.028)	0.960
PaO_2_/FiO_2_	0.993 (0.988-0.998)	0.003	0.997 (0.992-1.002)	0.207
APACHE II score	1.105 (1.061-1.151)	0.0001	1.070 (1.014-1.129)	0.013
WBC	1.042 (1.012-1.073)	0.006	0.904 (0.631-1.296)	0.584
PMN	1.052 (1.019-1.086)	0.002	1.194 (0.806-1.769)	0.377
Lactate	1.333 (1.194-1.489)	0.0001	1.204 (0.990-1.463)	0.063
PCT	1.003 (0.996-1.010)	0.433	NA	NA
Calreticulin	1.017 (1.002-1.031)	0.022	1.103 (0.991-1.034)	0.246

*p* values less than 0.05 were considered statistically significant. APACHE: Acute Physiology and Chronic Health Evaluation; CI: confidence interval; FiO_2_: inspiratory oxygen fraction; HR: hazard ratio; NA: not applicable; PaO_2_: arterial oxygen tension; PCT: procalcitonin; WBC: white blood cell; PMN: neutrophil; MAP: mean arterial pressure.

## Data Availability

The data used to support the findings of this study are available from the corresponding authors upon request.
